# Successful Full-Thickness Skin Regeneration Using Epidermal Stem Cells in Traumatic and Complex Wounds: Initial Experience

**DOI:** 10.7759/cureus.10558

**Published:** 2020-09-20

**Authors:** Arthur Berg, Sanjeev Kaul, Gregory E Rauscher, Melissa Blatt, Stephen Cohn

**Affiliations:** 1 Department of Trauma and Surgical Critical Care, Hackensack University Medical Center, Hackensack, USA; 2 Department of Trauma and Surgical Critical Care, Hackensack Univeristy Medical Center, Hackensack, USA

**Keywords:** stem cells, split thickness skin graft, full thickness skin graft, complex wounds, traumatic wounds, necrotizing fasciitis, crush injuries, degloving wounds, follicular bulge, biotechnology

## Abstract

Skin grafts generated from cultured autologous epidermal stem cells may have potential advantages when compared to traditional skin grafting. In this report, we will share our initial experience with a new technique for the treatment of difficult cutaneous wounds. Eight patients with traumatic or complex wounds underwent full-thickness skin harvesting and processing of epidermal stem cells, followed by the application of our novel management protocol. The patients were at high risk for non-healing and/or severe scar formation due to large traumatic de-gloving crush injuries, wounds from necrotizing fasciitis, or chronic wounds from osteomyelitis. We examined the percent graft success, recipient to donor size ratios, the median time to epithelialization, and two-point sensory discrimination. An international scale (The Patient and Observer Scar Assessment Scale - POSAS) was used to evaluate wound cosmesis and included parameters such as pain, pruritus, vascularity, pigmentation, and thickness of the healing wound. In total, 10 out of 11 wounds had 100% survival of the graft, and one patient had an 80% graft take. The largest wound was 1600 cm^2,^ and all wounds were harvested from small-donor sites, which were closed primarily. The mean wound to donor ratio was >25:1. Most wounds were fully epithelialized within 30 days. Neurologically, four out of six patients studied exhibited two-point discrimination similar to the adjacent native uninjured skin. The majority of patients reported their wounds to have limited pain or pruritus, and similar pigmentation to adjacent skin.

## Introduction

Major advancements in skin grafting did not occur until the 19th century with the advent of general anesthesia. The limitations of the commonly utilized split-thickness skin grafts are well described: wounds often develop severe scarring, hyperpigmentation, and/or hyperalgesia [1-3]. With the exception of autologous full-thickness skin grafts, no split-thickness or skin substitute has been able to fully replicate the architecture, function, or cosmetic appearance of native skin.

Stem cells have been shown to proliferate, differentiate, and survive when cultured, and may revolutionize the treatment of disease [4, 5]. A novel technique utilizing autologous epidermal stem cells may confer benefit in the coverage of major skin loss. Extensive skin constructs have been created from the harvesting of tiny portions of healthy skin prior to auto-grafting [2]. This homologous, autologous stem cell-derived graft material appears to not only accelerate wound healing but also to reinstate the native wound’s sensation, hair follicles, pigment, and glandular morphology [6]. We describe the first experience with ­­eight patients undergoing epidermal stem cell auto-grafting in the setting of complex wounds. There are no other case series in the literature describing the use of epidermal stem cells in the setting of traumatic or previously infected wounds.

## Case presentation

Eight patients presented to our institution with difficult to heal wounds. These included large traumatic wounds, debrided wounds from severe necrotizing fasciitis, and chronic wounds, which had failed prior skin grafting. Following our own novel protocol, a full-thickness segment of skin for harvest was removed from the thigh, groin, or abdomen. Care was taken to avoid cautery during graft harvest, so cellular morphology was not altered. The specimen was excised and placed in a sterile cup that contained a gentamicin antibiotic. The size of the graft was typically a small fraction of the size of the wound. The specimen donor site was then irrigated, and hemostasis was obtained prior to being closed primarily. The donor sites are all listed as one dimensional because they were closed primarily. 

**Figure 1:**
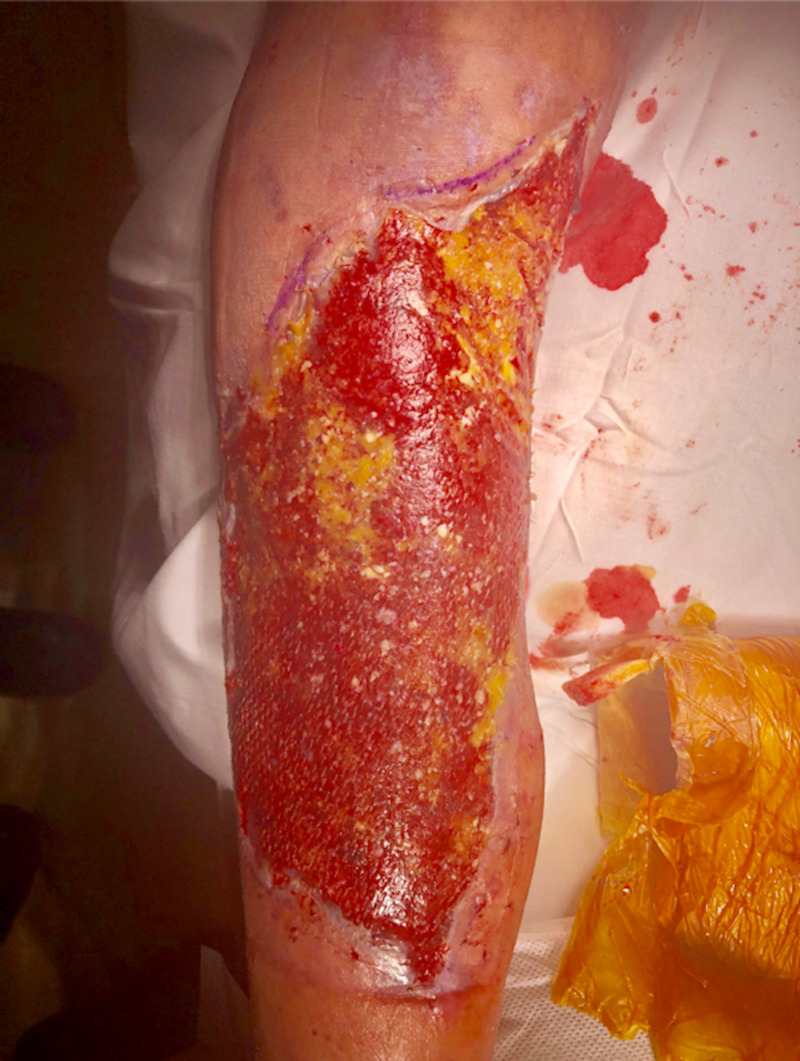
Dermal islands of growing tissue at two weeks

**Figure 2:**
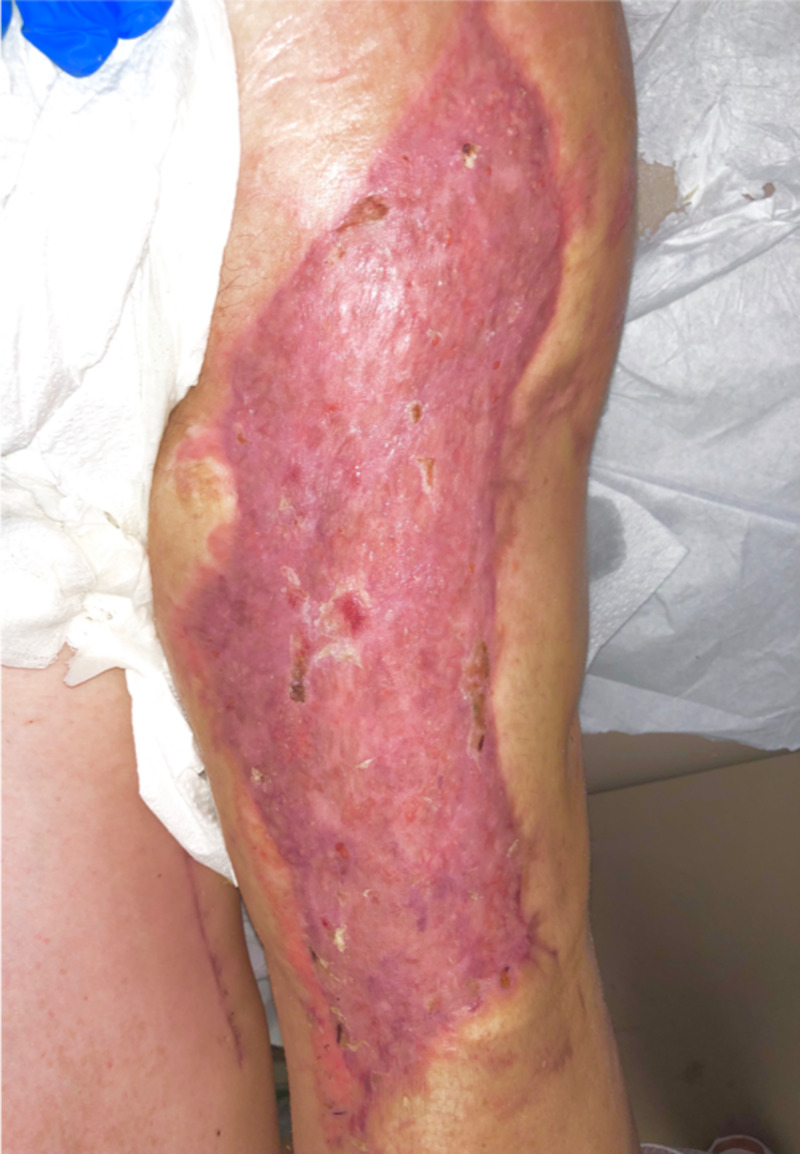
Fully functional, sensate, and glandular tissue at four weeks

After processing of the donor specimen at a bioengineering laboratory (Polarity TE, Salt Lake City, USA), the graft material was returned as a tissue paste in a sterile package. The recipient wound bed, at this point, had a clean bed of healthy granulation tissue. During the deployment, the graft material was spread on the wound bed as a paste similar in consistency to peanut butter. The wound was then covered by a thin sheet of silicone that was meshed in a 1.5:1 fashion using a Skin Graft Mesher (Zimmer® Surgical, Dover, USA) and placed directly over the graft material. The silicone sheet was secured by staples to the wound edge and then covered by a fibrin glue (Evicel™, Somerville, USA). Lastly, a white Granulofoam™ dressing (KCI, San Antonio, USA) was applied with an Ioban™ drape (3M™, St. Paul, USA) to secure the dressing. Negative pressure therapy was initiated and set to -75 mmHg. This dressing was then removed and reapplied every 5-7 days for three weeks. The dressing was then changed to a regular non-adherent material in between the wound and the overlying gauze bandage. Successful graft maturation was noted by the presence of dermal islands (see Figure 1). These were portions of healthy granulation that subsequently epithelialized and formed functional and glandular tissue (see Figure 2). The final graft resembled healthy skin by general appearance, pigmentation, and sensation. Additional images are provided in appendices.

The tissue was evaluated postoperatively in several ways. The Patient and Observer Scar Assessment Scale (POSAS) was used to evaluate the cosmesis and functionality of the wound. This assessment scale has been shown to have inter-observer reliability and is widely used amongst dermatologists and plastic surgeons worldwide [7]. It measures scar quality metrics, including pigmentation, pliability, thickness, and similarity to adjacent skin architecture. It is also unique in that it combines the patient’s viewpoint of the wound with the observer’s assessment. In addition to the POSAS evaluation, we assessed the grafts for two-point discrimination and time to epithelialization.

The average patient age was 46 years (see Tables 1-2). All patients were at high risk for non-healing and severe scar formation. Nearly all of the wounds were acute and treated during their initial hospitalization. In total, 10 out of 11 patients had 100% graft survival. The single patient who had only an 80% graft survival had been extensively and repeated debrided for Fournier’s gangrene of the perineum (Table 1, patient 7), and likely failed 100% grafting because of a flare-up of pyoderma gangrenosum. The largest wound was over 1600 cm^2, ^and all wounds were harvested from a donor site, which was closed primarily. The wound to donor ratio exceeded 25:1 on most patients. The average donor site scar was seven centimeters in length. All donor site scars were healed without pain or deformity. The average time to epithelialization of the graft recipient wound beds was 30 days. Neurologically, more than 50% of the patients exhibited two-point discrimination, which was similar to the adjacent native skin. Overall, patients reported the grafted wounds to be low in pain, pruritus and had similar pigmentation to adjacent skin.

**Table 1:** Wound characteristics LUE - left upper extremity; RUE - right upper extremity; LLE - left lower extremity; B/L - bilateral

Patient	Type/location	Wound/donor ratio	Two-point discrimination	Overall scar score	Percent take
1	Fournier’s gangrene	28:1	Yes	100%	100%
2	LUE traumatic degloving	37:1	Yes	70%	100%
3	RUE traumatic degloving	13:1	Yes	80%	100%
4	LLE traumatic degloving	75:1	Yes	50%	100%
5	Chronic osteomyelitis	10:1	No	100%	100%
6	Sacral ulcer and B/L heel	30:1	No	100%	100%
7	Fournier’s gangrene	18:1	Pending	Pending	80%
8	RUE traumatic degloving	22:1	Pending	80%	100%

**Table 2:** Demographics DM - diabetes mellitus; PVD - peripheral vascular disease; RSD - reflex sympathetic dystrophy

Patient	Type/location	Time to epithelization	Size of the wound	Harvest site	Size of donor site	Risk factors for poor wound healing
1: 48-year-old female	Necrotizing fasciitis - bilateral groin and perineum	3 weeks	Left groin: 20x8cm; right groin: 4x3cm	Right lateral thigh	6cm	DM
2: 56-year-old female	Traumatic degloving - left upper extremity	4 weeks	20x15cm	Left lateral thigh	8cm	none
3: 37-year-old male	Traumatic degloving - right upper extremity	8 weeks	8x5cm, 15x5cm	Right lateral thigh	8cm	none
4: 48-year-old female	Traumatic degloving - left lower extremity x2	4 weeks	50x20cm (thigh); 30x20cm (calf)	Right medial thigh, right groin	12cm (right thigh), 9cm (right groin)	none
5: 37-year-old male	Chronic osteomyelitis - right lower extremity	4 weeks	7x6cm	Lower abdomen	4cm	PVD, DM, RSD
6: 52-year-old male	Sacral ulcer and bilateral heel	4 weeks	Bilateral heel: 8x4cm; sacrum: 10x16cm	Lower abdomen	7cm	DM
7: 65-year-old male	Fournier’s gangrene - perineum	8 weeks	6x12cm	Lower abdomen	4cm	PVD, DM, pyoderma
8: 27-year-old male	Right upper extremity degloving	4 weeks	8x20cm	Right groin	7cm	none

## Discussion

The main findings of our study were: 1. epidermal stem cell skin grafts had a nearly 100% survival despite their use in highly complicated wounds; 2. the cosmetic appearance and functionality (including sensation) of the graft site was similar to that of adjacent native skin.

Split thickness skin grafts are commonly employed for coverage of various types of granulating wound beds, but have a number of limitations. These grafts are absent of normal sensation, and also lack the durability, appearance (i.e., pigmentation), and functionality of native skin [1, 3]. This is an inherent limitation of wound biology. Re-epithelization is limited by competing and opposing forces of fibroblasts during the proliferative phase of wound healing and is most apparent in large or deep wounds. This process promotes scar formation and secondary wound contracture without the functionality of normal skin [8]. Published literature has shown that high-risk patients with compromised wound biology have a high rate of failure (nearly 30%) with even the most advanced skin substitutes [6, 9]. On a cellular level, both chronic and complex wounds have dysregulated growth factors, cellular activity, and the persistence of a poorly differentiated state of keratinocytes [8].

**Figure 3:**
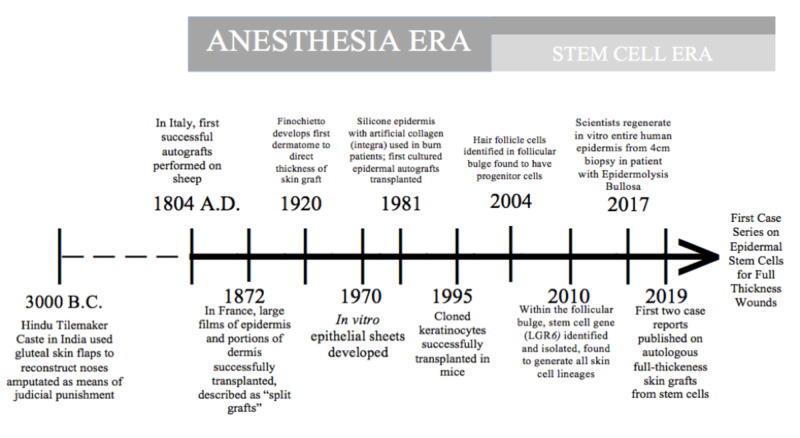
Timeline of advances in skin grafting

An increased understanding of the nature of stem cells, molecular signaling, and tissue engineering in the past two decades has made the clinical application possible (see Figure 3) [5]. Anatomically, the reservoir for epithelial stem cells lies in the outer root sheath in the proximal bulge of a hair follicle. Cell lineage studies performed by Ito and colleagues in 2004 showed that these pluripotent bulge cells and their progeny have the ability to proliferate and undergo terminal differentiation to form the native full-thickness architecture. This includes the hair shaft, melanin-producing cells, and glandular cells [10]. The same investigator was able to show, in animal studies, that these isolated bulge cells rapidly divide and migrate in the setting of the acute wound-healing response [11]. Radio-labeled leucine-rich repeat-containing G-protein coupled receptor, a transmembrane protein marker for stem cells, has been extensively studied and found throughout epithelial surfaces, specifically in the follicular bulge. In animal studies, these isolated cells were shown to have the ability to proliferate, migrate, and differentiate into native hair follicles [12].

A recent pediatric case report using the same skin construct from our investigation supported this cellular phenomenon. In this case, a biopsy of a child who had received the stem cell-derived graft five months prior demonstrated a normal distribution of melanocytes and structurally normal hair follicles. On further cell staining, this biopsy demonstrated a fully developed stratified epidermis with organized dermal plexus vasculature [2]. The ability for these pluripotent stem cells to differentiate has been studied extensively and is the result of precise signals from their microenvironment. This gives these stem cells the capacity to recognize, regenerate, and replace damaged or dying cells in their vicinity [5].

Our case series included only very complex wounds in a complicated patient population (Table 2). We focused on high risk, sometimes previously grossly infected, large wounds that traditional split-thickness skin grafting would have likely failed, and in two of our patients had failed. We feel that our excellent graft survival, along with the cosmesis and functionality of the grafts, would not have been possible without this epidermal stem cell-derived product. With regards to safety, the graft is autologous, so there does not appear to be a risk of immunogenic reactions.

There are a number of limitations to our investigation. The subjectivity of the scar assessment and time to epithelialization were apparent. The wounds were examined on follow up appointments that were spread apart by at least a week after discharge, limiting our analysis to periodic observations. The percent graft survival was somewhat subjective as well because it reflects a visual estimation of how well the graft took throughout the wound. This was not a major limitation because almost all the wounds had 100% graft survival. Our study is retrospective and limited by the size of the study population and, as a result, warrants further study. Additionally, there is no long-term data because our patients have only recently received grafting; however, this is the first report of its kind, evaluating a series of patients with complex wounds receiving successful grafting from epidermal stem cells. Future studies would include looking at tissue specimens and performing a skin biopsy of the recipient wounds beds and comparing them to adjacent native skin.

## Conclusions

We describe the index case series utilizing a novel tissue based, autologous, homologous epidermal stem cell derived product in complex surgical wounds. These wounds on average were very large and likely not amenable to traditional skin grafting. Despite requiring only a small full thickness donor skin specimen, more than 90% of the patients had a 100% graft survival covering large and complicated wound surfaces, and many experienced normal sensation at the healed graft site. Furthermore, our clinicians felt that cosmesis was far superior to the results seen with split thickness skin grafts. This is the first case series in the literature describing the use of epidermal stem cells. 

## Figures and Tables

**Figure 4:**
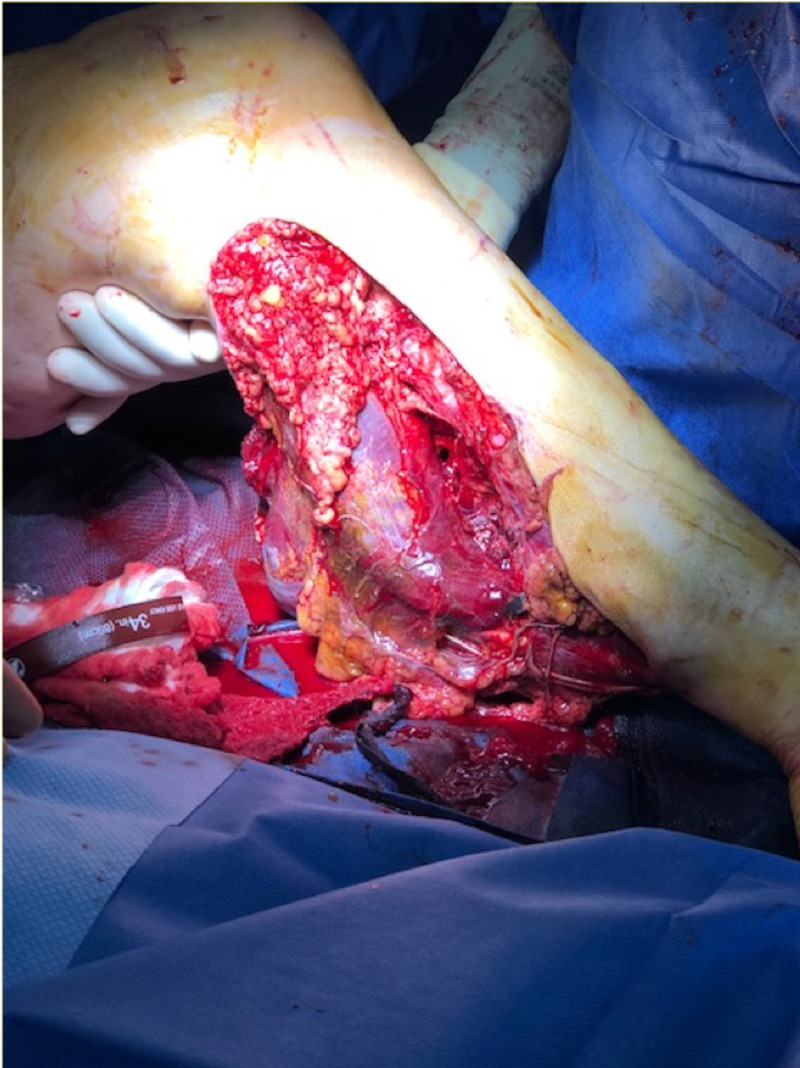
Traumatic degloving injury referenced in Figure 1

**Figure 5:**
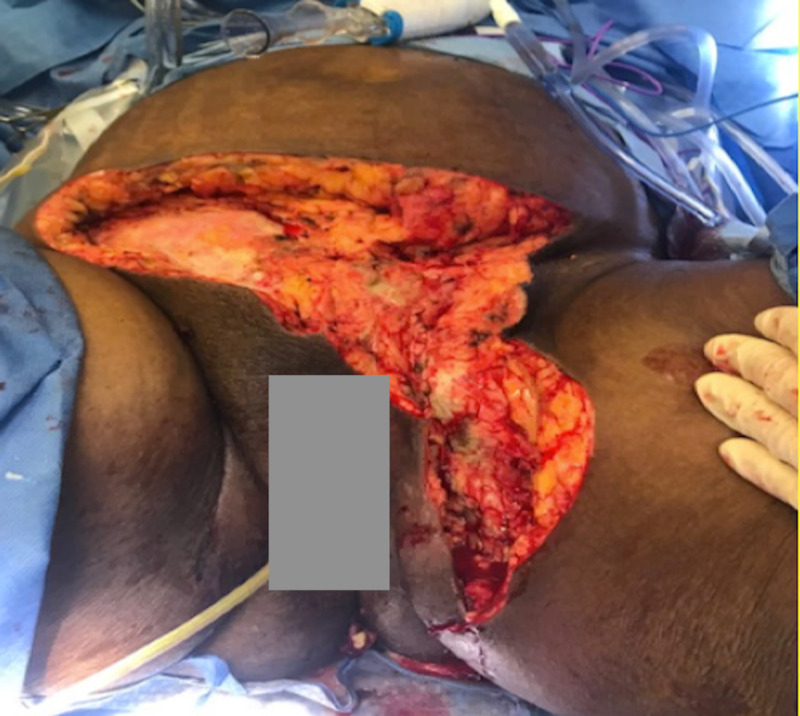
Large wound after multiple debridements from necrotizing fasciitis

**Figure 6:**
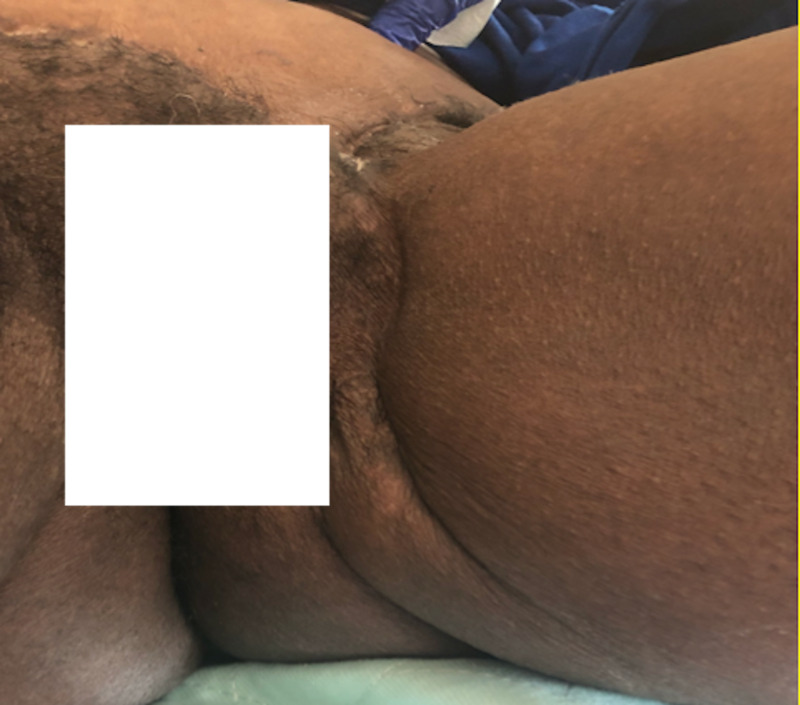
Previous patient several months after deployment of stem cell skin construct

**Figure 7:**
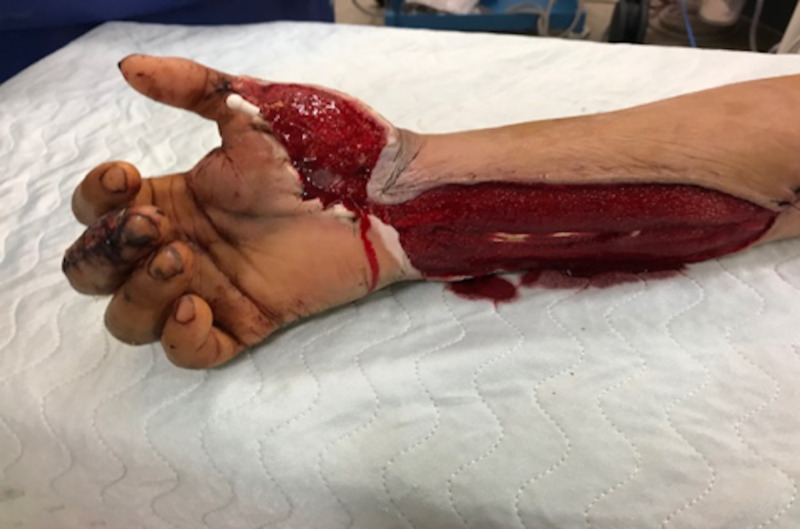
Traumatic degloving of right upper extremity (shown after multiple debridements)

**Figure 8:**
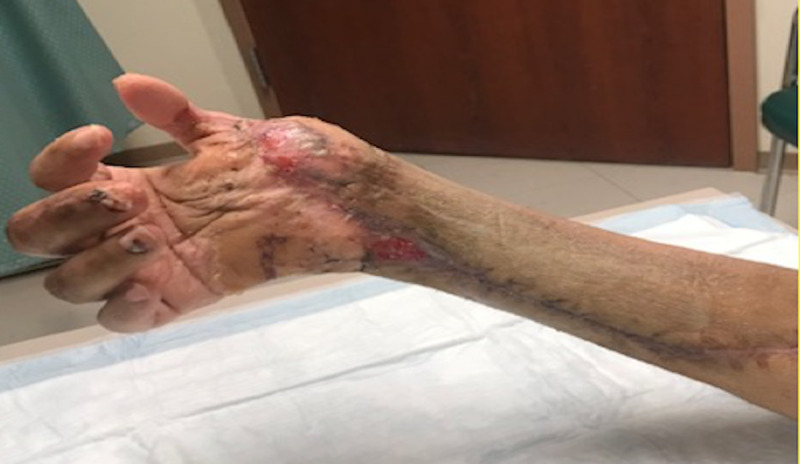
Previous patient shown weeks after deployment of stem cell skin construct
